# TACE Versus TARE in the Treatment of Liver-Metastatic Breast Cancer: A Systematic Review

**DOI:** 10.3390/tomography11070081

**Published:** 2025-07-12

**Authors:** Charalampos Lalenis, Alessandro Posa, Valentina Lancellotta, Marcello Lippi, Fabio Marazzi, Pierluigi Barbieri, Patrizia Cornacchione, Matthias Joachim Fischer, Luca Tagliaferri, Roberto Iezzi

**Affiliations:** 1 Radiology Unit, Bolzano Hospital, Azienda Sanitaria dell’Alto Adige, Via Lorenz Böhler 5, 39100 Bolzano, Italy; babis.lalenis@gmail.com; 2 Department of Diagnostic Imaging, Radiation Oncology, and Hematology, A. Gemelli University Hospital Foundation, IRCCS, Largo A. Gemelli 8, 00168 Rome, Italy; valentina.lancellotta@policlinicogemelli.it (V.L.); marcello.lippi01@icatt.it (M.L.); fabio.marazzi@policlinicogemelli.it (F.M.); pierluigi.barbieri@policlinicogemelli.it (P.B.); patrizia.cornacchione@policlinicogemelli.it (P.C.); luca.tagliaferri@policlinicogemelli.it (L.T.); roberto.iezzi@policlinicogemelli.it (R.I.); 3 Radiology Unit, Merano Hospital, Azienda Sanitaria dell’Alto Adige, Via G. Rossini 5, 39012 Merano, Italy; matthiasjoachim.fischer@sabes.it; 4 Faculty of Medicine and Surgery, Università Cattolica del Sacro Cuore, Sede di Roma, Largo F. Vito 1, 00168 Rome, Italy

**Keywords:** breast cancer, liver metastases, chemoembolization, radioembolization, systematic review

## Abstract

**Background/Objectives**: Liver metastases are common among patients with breast cancer and have a poor prognosis if left untreated. The aim of this systematic review is to evaluate and compare chemoembolization (TACE) versus radioembolization (TARE) treatments in patients with breast cancer liver-dominant metastases in terms of overall survival (OS), local tumor control (LC), and toxicity. **Methods**: The S.P.I.D.E.R framework was used to address the clinical question. A systematic literature search using PubMed and Scopus was performed to identify full articles evaluating the efficacy of TACE and TARE in patients with liver metastases from breast cancer. **Results**: The literature search resulted in 10 articles for TACE, 13 articles for TARE and 1 for combined TACE/TARE, totaling 462 patients for the TACE group and 627 for the TARE group. The median LC was 68.7% for TACE and 78.9% for TARE. The median OS was 15.3 months for TACE and 11.9 for TARE. Progression at three months was 32.5% for TACE and 20.6% for TARE. **Conclusions**: The included studies were heterogeneous, varying widely in design, patient selection, and therapeutic protocols. Nonetheless, this systematic review suggests that locoregional therapies are effective in the treatment of liver metastases in patients with breast cancer and may improve tumor burden, alleviate symptoms and extend overall survival. The median LC of the liver metastases at three months was higher in the TARE group compared to TACE. However, the TARE group showed lower OS rates after treatment.

## 1. Introduction

Breast cancer is the most diagnosed cancer among women and represents the second leading cause of cancer-related death among women worldwide, after lung cancer, and the first one among black and Hispanic women [1].

Breast cancer is divided into four molecular classes: Luminal A, Luminal B, HER2+, and Basal (or triple negative), based on the presence or absence of expression of the estrogen or progesterone genes and overexpression of the HER2 gene. This molecular classification has therapeutic implications for hormonal and anti-HER2+ therapies. Triple negative patients have the worst prognosis. Moreover, metastatic breast cancer is considered non-curable, even after the development of new therapies that target specific hormone receptors [2].

In breast cancer patients, the liver represents a common metastatic site and is the fourth most frequent site of distant metastasis (13.3%) after lymph nodes, bone, and lung [3]. The presence of hepatic metastases increases disease-related morbidity and mortality, being correlated with unfavorable outcomes [4]. In patients with untreated breast cancer liver metastases (BCLM), overall survival (OS) ranges from 4 to 8 months [5].

Systemic therapy represents the first treatment choice in this set of patients [6]. In liver-limited metastatic breast cancer, locoregional therapies have become a valid alternative to surgical resection, especially for patients with contraindications for surgery or for lesions with unfavorable anatomical positions for surgical approach [7]. In patients with hepatic oligometastatic disease progression, or in those in which systemic therapy has failed or is not well-tolerated, transarterial locoregional therapies like chemoembolization (TACE) and radioembolization (TARE) can be helpful, conferring a survival advantage [7]. Moreover, locoregional treatments, by inducing tumor necrosis, release tumor antigens in the bloodstream, which may trigger anti-tumoral immune response [7].

TACE and TARE are based on intra-arterial super-selective administration of chemotherapic drugs and radioisotopes, respectively, via the tumor-feeding artery, to obtain a local therapeutic effect [8]. These treatments are currently acknowledged as one of the mainstays in the treatment of hepatocellular carcinoma [8]. Intra-arterial procedures like TACE and TARE could represent an alternative treatment option for patients with breast cancer liver metastases, also considering that patients with non-cirrhotic livers could benefit from higher therapeutic doses of radioisotopes during TARE compared to hepatocellular carcinoma. However, although these options are applied in clinical practice in breast cancer patients with liver metastases, they are not depicted in guidelines [9]. Based on this background, the aim of this study is to compare survival outcomes, response rates and toxicity in patients with metastatic liver disease from breast cancer who underwent TACE or TARE.

## 2. Materials and Methods

### 2.1. Development of Clinical Questions

The S.P.I.D.E.R. (Sample, Phenomena of Interest, Design, Evaluation, Research type) framework was used and is reported in Table 1 [10].

### 2.2. Identification of Outcomes

The following outcomes of benefit were identified: local control and overall survival. Local control was defined as the time interval between the date of TACE or TARE start and the date of “in situ” thermal ablation field relapse/progression/persistence of disease or the date of the last follow-up. Overall survival was defined as the time interval between the date of TACE or TARE start and the date of death or last follow-up. The following outcomes of harm were identified: acute and late toxicities grade III–IV. All these outcomes were considered as “critical” for the decision-making process.

### 2.3. Search Strategy and Selection of Evidence

Systematic research using PubMed and Scopus was performed to identify full articles evaluating the efficacy of TACE and TARE in patients with liver metastases from breast cancer. The studies were identified via advanced searches using medical subject headings (MeSH), as follows: (chemoembolization) (“breast neoplasms” [MeSH Terms] OR (“breast” [All Fields] AND “neoplasms” [All Fields]) OR “breast neoplasms” [All Fields] OR (“breast” [All Fields] AND “cancer” [All Fields]) OR “breast cancer” [All Fields]) AND (“chemoembolic” [All Fields] OR “chemoembolisation” [All Fields] OR “chemoembolisations” [All Fields] OR “chemoembolism” [All Fields] OR “chemoembolization” [All Fields] OR “chemoembolizations” [All Fields] OR “chemoembolized” [All Fields]) AND (radioembolization) AND (breast cancer [MeSH Terms]) (“radioembolic” [All Fields] OR “radioembolisation” [All Fields] OR “radioembolization” [All Fields] OR “radioembolizations” [All Fields]) AND “breast neoplasms” [MeSH Terms] (breast cancer).

We also performed PubMed and Scopus searches with the following keywords: “chemoembolization breast cancer liver metastases” and “radioembolization breast cancer liver metastases”. Searches were restricted to the English language. A timeframe from 1994 to 2024 as years of publication was considered for PubMed, while from 2014 to 2024 for Scopus. We only analyzed full-text papers of clinical studies on patients with BCLM treated with TACE or TARE. Conference papers, conference abstracts, surveys, letters, editorials, book chapters, and reviews were excluded.

Three independent authors (CL, ML, FM) screened citations in titles and abstracts to identify valuable papers. Uncertainties about inclusion in the review were considered by an expert team (AP, VL, PB). Finally, a multicentric multidisciplinary committee (MF, RI, LT) performed an independent check and definitive approval of the review.

Inclusion criteria were:

1. Randomized controlled trials (RCTs), prospective, retrospective, and cohort studies;
2. BCLM patients with tumor progression under prior systemic chemotherapy and subsequent treatment with TACE or TARE;
3. Reported quantitative outcome data.

**Figure 1:**
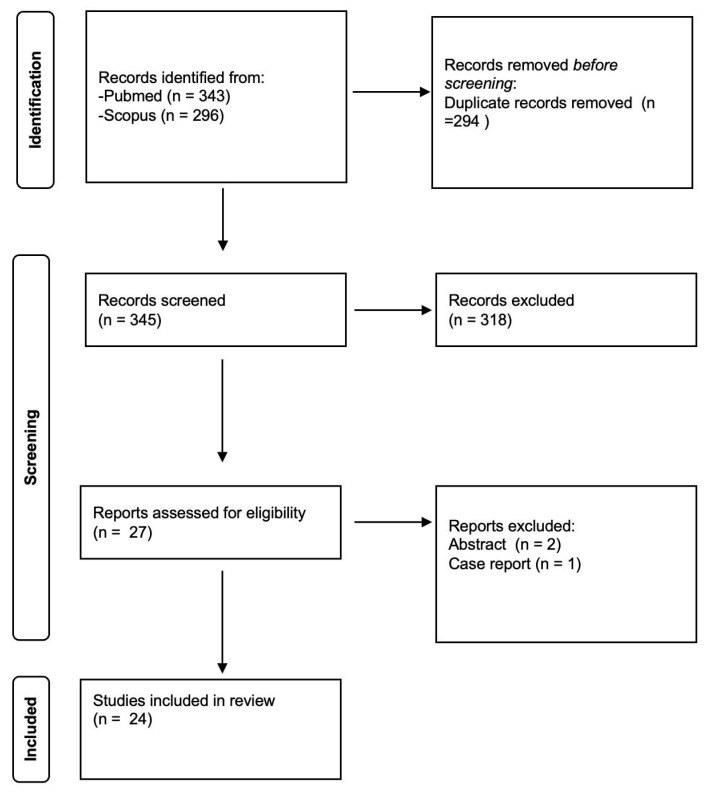
PRISMA flowchart of study selection.

The GRADEpro Guideline Development Tool (GDT) was used to create table summaries of results in Cochrane systematic reviews, considering study limitations, imprecision, indirectness, inconsistency, and publication bias [11]. Quality assessment revealed a high degree of clinical and methodological heterogeneity among the included studies, so quantitative synthesis was not useful. Therefore, meta-analysis was not performed. The Preferred Reporting Items for Systematic Reviews and Meta-Analyses (PRISMA) standards were used in order to conduct this systematic review (Figure 1).

### 2.4. Procedural Technique

#### 2.4.1. TACE

TACE is performed by injecting the chemotherapeutic drug into liver tumors, with an embolic effect and arterial occlusion in addition to molecular suppression of tumor growth [7].

The intra-arterial delivery of chemotherapeutic drugs into liver hepatic lesions can be performed in a selective or lobar mode. Most of the time, the femoral artery is catheterized using the Seldinger technique and an angiographic survey of the superior mesenteric artery (SMA) and celiac trunk is performed. As an alternative, transradial approach can be used, particularly for lobar procedures [12]. Once portal vein patency is confirmed by SMA angiography, a catheter is placed into the hepatic artery and then, often using a microcatheter, the segmental branches of the hepatic artery are catheterized, and chemoembolization is performed. For patients with bilobar disease, treatment should be performed to control the lobe with the higher tumor burden as seen by imaging performed immediately before the procedure; the second lobe should be handled in another session. In the studies included in our systematic review, various chemoembolization schemes were used, with different chemotherapeutic drugs such as cisplatin, doxorubicin, mitomycin, gemcitabine, and adriamycin, combined with Lipiodol or drug-eluting beads (DEBs).

#### 2.4.2. TARE

TARE consists of the injection of a β-emitting isotope, usually Yttrium-90 (Y90), integrated either inside the glass matrix of the microspheres (in case of Therasphere, Boston Scientific, Boston, MA, USA) or on the surface of the resin microspheres (in case of Sir-Spheres, Sirtex Medical, Sydney, Australia) [13,14]. TARE represents a new concept of transarterial interventional radiotherapy that allows concentrated β-radiation administration to tumor tissues while minimizing damage to the surrounding liver parenchyma [15]. Preprocedural planning currently relies on infusion of Technetium-99m (99mTc) macroaggregated albumin (MAA) into the hepatic artery, followed by thoracic and abdominal perfusion scintigraphy to rule out substantial hepatopulmonary shunting. If the hepatopulmonary shunt exceeds 20% of the applied 99mTc-MAA activity, the procedure should not be performed due to the high risk of radiation-related lung injury [16]. Prophylactic embolization (usually with metallic coils) of hepatoenteric arteries that originate distal to the injection site or close to it is advisable to prevent non-target embolization and avoid severe gastrointestinal complications such as gastric and duodenal ulcers, which are often difficult to manage, increasing morbidity and mortality among TARE patients [17,18]. However, in their systematic review, Borggreve and colleagues did not find any differences in terms of complications in patients who underwent gastroduodenal artery, right gastric artery, cystic artery, or hepatic falciform artery embolization compared to patients that did not have prophylactic embolization [19].

## 3. Results

The literature search resulted in 24 final studies included in this review; 10 TACE studies, 13 TARE and one combined TACE/TARE study fulfilled the inclusion criteria and were included in this review. A total of 462 patients were included in the TACE group [20,21,22,23,24,25,26,27,28,29,30]. A total of 601 patients were included in the TARE group [28,31,32,33,34,35,36,37,38,39,40,41,42,43].

### 3.1. TACE Group

Eight studies were retrospective [20,21,22,23,24,28,29,30]. Three studies were prospective [25,26,27]. Among the 462 patients treated with TACE, 78.6% (363/462) had liver-only disease, while 21.4% (162/462) had extrahepatic disease, the majority with bone localizations. The liver disease distribution was noted in 3/9 studies: unilobar disease in 49% of cases (37/75) and bilobar in 51% of cases (38/75).

In terms of TACE protocol, heterogeneous treatment schemes are reported: polyvinyl-alcohol (PVA) particles were used in six studies (54.5%), lipiodol plus microparticles in four studies (36.4%), and absorbable gelatin sponge was used in one study (9.1%). The microparticles were also quite heterogeneous in size (range 70–560 microns). Doxorubicin was used in five studies (45.5%); in particular, it was used as the only chemotherapeutic agent in 3/11 studies (27.3%), while it was used in combination with other agents in 2/11 studies (18.2%). The remnant studies (54.5%) utilized various other therapeutic schemes.

Table 2 summarizes the study findings together with the therapeutic schemes.

Follow-up time was not reported in 5/11 studies (45.5%). Six studies (54.5%) reported a mean follow-up time of 18 months (range 4–29 months).

Treatment response evaluation was performed with computed tomography (CT), magnetic resonance imaging (MRI), and/or positron emission tomography (PET)-CT examinations, using the Response Evaluation Criteria In Solid Tumors (RECIST), modified RECIST (mRECIST), and PET Response Criteria In Solid Tumors (PERCIST) criteria.

One study reported a one-month overall response rate (ORR, given by CR + PR) of 74.1% (20/27 patients), stable disease (SD) of 22.2% (6/27 patients), overall disease control (ODC, given by CR + PR + SD) rate of 96.3% (26/27 patients), and progressive disease (PD) rate of 3.7% (1/27 patients) [30].

The other 10 studies (90.9%) reported a three-month follow-up ORR of 37.6% (range 7–71.4%), a SD of 31% (range 7–57%). The three-month median ODC was 68.7% (range 31–92.8%), while the three-month PD was 30.3% (range 7.2–60%).

The median OS of ten studies, calculated from treatment to patient’s death, was 15.3 months (range: 6–28 months). One study did not report medial OS [24].

Post-embolization syndrome was the most frequent adverse event and usually required only symptomatic treatment. Complications were reported according to Clavien–Dindo score in nine studies: the grade ≥ III complication rate was 8.3% (range 0–35%) [44]. Histological characteristics of the tumor were known in eight studies (72.7%).

### 3.2. TARE Group

Two studies were prospective [33,36]. All twelve other studies were retrospective [31,32,34,35,37,38,39,40,41,42,43].

One study did not mention the presence or absence of extrahepatic disease [31].

Among the other studies, 33% (199/601) of patients had liver-only disease, while 67% (402/601) had extrahepatic disease, the majority with bone localizations. The liver disease distribution was described in 10/14 studies: unilobar in 30% of cases (135/447) and bilobar in 70% of cases (312/447).

In terms of TARE protocol, 100% of authors used Yttrium-90 (90Y), loaded into different microspheres; in particular, resin microspheres (SIR-spheres) were used in 7/14 studies, glass microspheres (Theraspheres) were used in 3/14 studies and a combination of both in 4/14 studies. Follow-up time was not reported in 6/14 studies (42.9%). Eight studies (57.1%) reported a mean follow-up time of 14.7 months (range 4–24 months).

One study mentioned local control with PERCIST criteria obtaining an Objective Response (OR) of 46% [43]. Another study classified 52% of patients as responders and 48% as non-responders based on 18F-FDG PET, defining PET response as a greater than 30% decrease in SUVmax from baseline to follow-up determined in up to five lesions [34]. Two studies did not report results on local control [36,37].

The remaining studies showed the following results: at three months the median ORR (CR + PR) rate was 39.4% (range 9–61%), SD rate was 39.5% (range 6.7–52%), ODC (CR + PR + SD) rate was 78.9% (range 46.7–96%), while the three-month median PD rate was 20.7%. The median OS was 11.9 months (range 4.9–35.4 months). Grade ≥ III complications were reported in 49/580 patients (8.5%, range 2.3–16%). Histological characteristics of the tumor were known in only nine studies [28,32,33,34,35,37,38,39,43].

Table 3 summarizes the principal findings of each study.

### 3.3. Comparison Between TACE and TARE

TACE and TARE ORR rates did not differ (37.6% versus 39.4%), even though ODC rates were in favor of TARE (78.9% versus 68.7%). Moreover, the median three-month PD rate was higher for TACE (30.3%) than TARE (20.7%). In terms of OS rates, there was a longer survival in the TACE group (15.3 months) versus the TARE group (11.9 months). Regarding grade ≥ III complication rates, there were no differences between the two groups.

## 4. Discussion

Establishing the most appropriate treatment for breast cancer patients with liver metastases, while maintaining an adequate quality of life and obtaining optimal response, represents a strong challenge. Surgical resection is associated with excellent response rates, but the number of patients that are eligible for resection is small [45]. The use of systemic chemotherapy remains the standard of care. However, response rates with chemotherapeutic treatments vary from 11.6% to nearly 83% [46,47].

TACE and TARE represent a valid therapeutic option in chemorefractory patients or in patients in which chemotherapy is deemed unfeasible. This is due to TACE and TARE’s effectiveness and safety, even though data are limited to non-randomized studies. Despite these limitations, TACE and TARE have several advantages. This systematic literature review shows that TACE and TARE represent valid treatment options in breast cancer patients with liver metastases, without prohibitive toxicity and with good results in terms of OS and ODC. In particular, according to the studies included in this systematic review, TARE provides significantly better ODC compared to TACE, with similar rates of grade ≥ III complications. This difference is mainly due to the higher rates of SD in the TARE group, which may be a consequence of better clearance of non-visible metastatic foci performed by TARE. This could be due to TARE’s greater cytotoxic effect on tumoral cells compared to TACE’s hypoxia plus local chemotherapy effect [48]. These results are encouraging, especially considering the performance status of these patients and that they have often already undergone previous treatments.

On the other hand, the less favorable TARE results in terms of OS could presumably be related to the greater extrahepatic tumor burden in TARE patients and, at least to a small degree, to the greater hepatotoxic effect of radiation in this type of treatment upon normal hepatocytes. This urges the need for proper patient selection prior to treatment with TARE, with particular interest in liver function. It is very important to perform laboratory tests and adequate clinical evaluation, optimistically in a multidisciplinary tumor board setting, in order to select the right group of patients who could benefit from these treatments.

For comparison, a 2021 systematic review by Aarts and colleagues reported similar results between TACE and TARE in terms of OS and LC [49]. However, there are some differences between the 2021 systematic review and this one, as this study also includes four more recent studies. Moreover, chemo-infusion was not included in the search criteria of our systematic literature search.

From evaluation of the included studies, various data emerge regarding the role of different factors related to patient, tumor and technique in the success or failure of these procedures. In particular, in a study by Eichler and colleagues it was reported that strongly vascularized tumors were associated with significantly lower response rates compared to tumors with moderate vascularization (*p* = 0.0028) [25]. On the other hand, various studies reported that lymph node status of the primary cancer, clinical stage of liver metastases, Child–Pugh grade, loss of weight, primary tumor size, hormone status of primary tumor (positive versus negative), disease-free interval, number of liver metastases, combined treatment methods (TACE plus chemotherapy, mitomycin C plus gemcitabine), and metastasis location (intra- or extrahepatic) were associated with better OS in univariate analysis [21,22,24,30]. Duan and colleagues reported a significant correlation between tumor histology and median OS after TACE for patients with positive estrogen receptor (ER+) status (30 months) versus those with negative ER (ER–) status (18 months) [24]. Except for hormone status, these factors were also independent factors in multivariate analysis [24].

In a study from Pieper and colleagues, in univariate analysis, the baseline eastern cooperative oncology group performance status (ECOG-PS) and treatment response based on RECIST criteria at three-month follow-up was correlated with the time to extrahepatic progression. Univariate analysis of OS showed significant interrelationships with baseline ECOG-PS, histology of the primary tumor, tumor vascularity, estimated relative liver tumor burden, occurrence of extrahepatic progression, chemotherapy after radioembolization, and baseline alanine aminotransferase (ALT), aspartate aminotransferase (AST), and gamma-glutamyltransferase (GGT) levels. On multivariate analysis, only baseline ECOG-PS (Eastern Cooperative Oncologic Group Performance Status) and baseline GGT levels were identified as independent predictors of OS [35].

A study by Schatka and colleagues showed that in univariable Cox regression, higher ECOG performance status, the presence of any baseline transaminase elevation with Common Terminology Criteria for Adverse Events (CTCAE) grade ≥ 2, prior surgical treatment, and a dose reduction for TARE were each significantly associated with shorter OS (*p* < 0.05). In multivariable analysis, ECOG ≥ 1 and the presence of any baseline transaminase with CTCAE grade ≥ 2 remained significant predictors of shorter OS, while prior liver surgery and dose reduction were not significant (*p* > 0.1). Baseline transaminase levels and better performance status were related with statistically significantly better OS (median OS 19.2 months versus 2.2 months) [37].

Fendler and colleagues reported that transaminase toxicity grade of 2 or more (*p* = 0.009) and tumor-to-liver ratio of 50% or more (*p* < 0.001) were independently associated with significantly reduced OS after TARE [34].

Currently, transarterial chemoembolization (TACE) and TARE are not included in major international clinical guidelines—such as those from the ESMO or the National Comprehensive Cancer Network (NCCN)—for the treatment of liver metastases from breast cancer [9,50]. For instance, the NCCN guidelines do not list TACE or TARE as a recommended option for hepatic metastases in breast cancer patients. However, they are sometimes considered in clinical practice as a palliative or symptomatic treatment for selected patients, particularly those with liver-dominant disease that is refractory to systemic chemotherapy. According to the results obtained from this systematic review, TACE and TARE for breast cancer liver metastases seem to have a quite low ≥ grade 3 complication rate and good OS and ODC rates. Multiple studies included in the review identified prognostic factors associated with improved outcomes. These include positive estrogen receptor status (ER+), moderate tumor vascularity, absence of extrahepatic disease, good baseline ECOG-PS, and normal baseline liver function tests (transaminases and gamma-glutamyltransferase). Importantly, ECOG performance status and baseline GGT levels were consistently identified as independent predictors of OS in multivariate analyses.

Limitations of this systematic review are represented by the retrospective nature of the majority of the included studies, as well as the small sample size of all studies and the short median follow-up. Furthermore, the studies are very heterogeneous, especially those on TACE, due to the use of different carriers (lipiodol and various microparticles), as well as due to the use of different chemotherapeutic drugs. There is also population inhomogeneity between TARE and TACE studies; in particular, patients subjected to TARE had a greater rate of extrahepatic disease, 67% vs. 30%. Another limitation of this systematic review is that of having heterogeneous data for the criteria used to evaluate the effectiveness of the treatment (PERCIST, mRECIST, RECIST). These limitations reflect the heterogeneity of indications for TACE and TARE in the treatment of breast cancer liver metastases. In our experience, these treatments have been performed with encouraging results in selected patients with oligometastatic liver-dominant disease, in particular when curative and systemic treatments were not feasible.

Due to the lack of prospective randomized trials, breast cancer patients with liver metastases should be enrolled in prospective studies. To improve the benefits of technical advances, all patients should be discussed by a multidisciplinary board [51]. Improved clinical outcomes and more uniform treatment approaches were made possible by professional multidisciplinary team discussions of clinical cases [52]. Finally, it is important to stress the necessity of combining treatment outcomes from many facilities in order to develop prediction models, as they have been shown to be a very helpful tool in patient counseling [53,54].

## 5. Conclusions

Although the studies included in our systematic review vary widely in design, patient selection (also including baseline differences in terms of extrahepatic disease burden), and treatment and follow-up protocols, the results suggest that locoregional therapies are effective in the treatment of liver metastases in patients with breast cancer and may help reduce tumor burden, alleviate symptoms, and extend local disease control and overall survival.

## Figures and Tables

**Table 1:** S.P.I.D.E.R. framework.

Item	Explanation
Sample	Breast cancer liver metastases
Phenomena of Interest	TACE and TARE
Design	Named types of qualitative data collection and analysis
Evaluation	LC, OS, complications
Research type	Qualitative method

Legend: TACE: transarterial chemoembolization. TARE: transarterial radioembolization. LC: local control. OS: overall survival.

**Table 2:** Studies implementing TACE in BCLM patients.

Author	Period	Study Type	Treated Patients	Treatment	LC at 3 Months	OS (Months)	Embolic Agent	Toxicity (Grade ≥ III)	Follow-Up (Months)
Giroux [20]	1994–2000	Retrospective	8	TACE after CHT failure	ORR 62% SD 12% PD 26%	6	Cisplatin, doxorubicin, mitomycin C + Lipiodol and PVA	N/A	N/A
Li [21]	1995–2000	Retrospective	28	TACE post-CHT	ORR 35.7% SD 46.4% PD 17.9%	28	Doxorubicin + Lipiodol	0%	28
Vogl [22]	1998–2006	Retrospective	208	159 TACE 49 TACE+ CHT	ORR 13% SD 50.5% PD 36.5%	18.5	Mitomycin C, gemcitabine + Lipiodol and microspheres	0%	N/A
Cho [23]	1998–2008	Retrospective	10	TACE	ORR 20% SD 20% PD 60%	12	Adriamycin, cisplatin, gemcitabine, oxaliplatin	0%	N/A
Duan [24]	1998–2008	Retrospective	44	44 TACE + CHT	ORR 59.1% SD 25% PD 15.9%	N/A	5FU/5FUDR, cisplatin, doxorubicin + Lipiodol, gelsponge	2.3%	29
Eichler [25]	N.A.	Prospective	43	TACE	ORR 7% SD 39% PD 54%	10.2	Gemcitabine + Lipiodol, DSM	11%	4
Lin [26]	2012–2014	Prospective	23	TACE	ORR 26% SD 57% PD 17%	17	Doxorubicin + DEBs	35%	N/A
Martin [27]	2007–2011	Prospective	40	TACE	ORR 58% SD 32% PD 10%	15	Doxorubicin + DEBs	3%	12
Chang J [28]	2006–2016	Retrospective	17	TACE	ORR 24% SD 7% PD 58% Unk 11%	4.6	Doxorubicin + DEBs	9%	9
Chang X [29]	2021	Retrospective	14	TACE	ORR 71.4% SD 21.4% PD 7.2%	20	Epirubicin, gemcitabine + DEBs	N/A	N/A
Zhao [30]	2010–2016	Retrospective	27	TACE	N/A (1-month LC)	22	Pirarubicin + gelsponge	14.8%	26

Abbreviations: LC: local control; OS: overall survival; TACE: transarterial chemoembolization; CHT: chemotherapy; ORR: overall response rate; SD: stable disease; PD: progressive disease; PVA: polyvinyl alcohol particles; DSM: degradable starch microspheres; DEB: drug eluting beads; Unk: unknown status.

**Table 3:** Studies implementing TARE in BCLM patients.

Author	Period	Study Type	Treated Patients	Treatment	LC at 3 Months	OS (Months)	Embolic Agent	Toxicity (Grade ≥ III)	Follow-Up (Months)
Bangash [31]	2007	Retrospective	27	TARE	ORR 39% SD 52% PD 9%	4.9	Glass microspheres	15%	N/A
Coldwell [32]	2002–2005	Retrospective	44	TARE	ORR 47% SD 47% PD 6%	14	Resin microspheres	16%	14
Jakobs [33]	2003–2007	Prospective	30	TARE	ORR 61% SD 35% PD 4%	11.7	Resin microspheres	13%	14.2
Fendler [34]	2003–2013	Retrospective	81	TARE	Responders 52% Non-responders 48%	8.75	Resin microspheres	10%	N/A
Pieper [35]	2006–2015	Retrospective	44	TARE	ORR 28.9% SD 42.2% PD 28.9%	6.13	56 Resin, 13 Glass microspheres	2.3%	4
Helmberger [36]	2014–2020	Prospective	47	TARE	N/A	10.6	Resin microspheres	N/A	24
Schatka [37]	2016–2021	Retrospective	38	TARE	N/A	6.4	Resin microspheres	5%	N/A
Chang [28]	2006–2016	Retrospective	30	TARE	ORR 40% SD 6.7% PD 50% Unk: 3.3%	12.9	49 Resin, 3 Glass microspheres	0%	9
Barakat [38]	2010–2019	Retrospective	31	TARE	ORR 46.7% SD 23.3% PD 30%	13	Glass microspheres	9.4%	N/A
Davisson [39]	2013–2018	Retrospective	24	TARE	ORR 9% SD 52% PD 39%	35.4	19 Resin, 4 Glass, 1 Resin + Glass microspheres	16%	22.3
Saxena [40]	2006–2012	Retrospective	40	TARE	ORR 32% SD 39% PD 29%	13.6	Resin microspheres	0%	11.2
Gordon [41]	2001–2013	Retrospective	75	TARE	ORR 35.3% SD 63.2% PD 1.5%	6.6	Glass microspheres	7.6%	N/A
Cianni [42]	2005–2011	Retrospective	52	TARE	ORR 55% SD 35% PD 10%	11.5	Resin microspheres	3%	N/A
Ridouani [43]	2011–2019	Retrospective	64	TARE	OR 46% (PERCIST)	10.9	22 Resin, 42 Glass microspheres	12.5%	19.1

Abbreviations: LC: local control; OS: overall survival; TARE: transarterial radioembolization; ORR: overall response rate; SD: stable disease; PD: progressive disease; OR: objective response.

## Data Availability

Data are available on request.

## Abbreviations

The following abbreviations are used in this manuscript:

TACE	Transarterial Chemoembolization
TARE	Transarterial Radioembolization
OS	Overall Survival
LC	Local Control
SPIDER	Sample, Phenomena of Interest, Design, Evaluation, Research type
BCLM	Breast Cancer Liver Metastases
MeSH	Medical Subject Headings
GDT	GRADEpro Guideline Development Tool
PRISMA	Preferred Reporting Items for Systematic Reviews and Meta-Analyses
SMA	Superior Mesenteric Artery
DEBs	Drug-Eluting Beads
Y90	Yttrium-90
99mTc	Technetium-99m
MAA	Macroaggregated Albumin
PVA	Polyvinyl Alcohol
CHT	Chemotherapy
ORR	Overall Response Rate
SD	Stable Disease
PD	Progressive Disease
DSM	Degradable Starch Microspheres
Unk	Unknown
CT	Computed Tomography
MRI	Magnetic Resonance Imaging
PET	Positron Emission Tomography
ODC	Overall Disease Control
OR	Objective Response
ER	Estrogen Receptor
ALT	Alanine Aminotransferase
AST	Aspartate Aminotransferase
GGT	Gamma-Glutamyl Transferase
ECOG-PS	Eastern Cooperative Oncology Group Performance Status
CTCAE	Common Terminology Criteria for Adverse Events
